# Coupling between stomatal conductance and photosynthesis of subtropical tree seedlings under warming and progressive drought

**DOI:** 10.3389/fpls.2026.1846577

**Published:** 2026-06-29

**Authors:** Zhenzhen Yuan, Xuming Wang, Yao Li, Qi Liu, Suimin Huang, Lei Li, Xiao Ying Gong

**Affiliations:** 1 Key Laboratory for Humid Subtropical Eco-geographical Processes of the Ministry of Education, School of Geographical Sciences, Fujian Normal University, Fuzhou, China; 2 National Field Observation and Research Station (Fujian Sanming) for Forest Ecosystem, Sanming, China; 3 Fujian Provincial Key Laboratory for Plant Eco-physiology, Fuzhou, China

**Keywords:** carbon uptake, climate change, drought sensitivity, elevated temperature, mesophyll conductance, progressive drought

## Abstract

Climate warming intensifies drought stress, threatening the carbon sink capacity of forest ecosystems. The coupling between stomatal conductance (*g*_s_) and photosynthesis (*A*) is crucial for predicting carbon cycling, yet its stability under combined warming and drought remains uncertain. We conducted a controlled experiment using open-top chambers to simulate warming (+1°C) combined with a progressive drought on seedlings of three dominant subtropical tree species (*Castanopsis carlesii*, *Schima superba*, and *Cunninghamia lanceolata*) in China. The responses of leaf gas exchange and chlorophyll fluorescence to these stressors were examined. Our results show that warming did not increase photosynthetic sensitivity to progressive drought across species. Despite significant drought-induced declines in photosynthesis, *g*_s_ and *A* remained tightly coupled. Leaf photosynthesis was mainly limited by reduced CO_2_ diffusion (decrease in both *g*_s_ and mesophyll conductance); meanwhile, there was no clear indication of photochemical damage. Species diverged in stomatal regulation: *C. carlesii* exhibited “profligate” stomatal behavior (higher slope parameters (*m*_1_) from the unified stomatal optimization model), while *S. superba* and *C. lanceolata* were conservative (lower *m*_1_). This study shows that these subtropical trees can maintain leaf-level *A*-*g*_s_ coupling during moderate warming and progressive drought, and the use of species-specific stomatal parameters is recommended when modeling carbon and water flux in subtropical forests.

## 1 Introduction

Global warming intensifies the severity and frequency of droughts (IPCC, 2021; Gebrechorkos et al., 2025), exacerbating plant mortality and threatening terrestrial vegetation carbon sequestration (Adams et al., 2009; Mahecha et al., 2022). Understanding plant physiological responses to combined warming and drought becomes crucial for accurately assessing gross primary productivity (GPP). Currently, land surface models estimate GPP by assuming coupled stomatal conductance (*g*_s_) and net assimilation rate (*A*) (Ball et al., 1987; De Kauwe et al., 2013). However, this assumption is challenged under compound climatic stresses. Though the combined effects of warming and short−term or severe droughts have been studied (Wang et al., 2026), how warming superimposed on progressive drought affects the *g*_s_–*A* relationship remains uncertain. Two opposing possibilities exist: warming could exacerbate water stress and force tighter stomatal closure to avoid hydraulic failure; alternatively, plants might keep stomata relatively open to enhance transpirational cooling. Which strategy prevails can be dependent on the environment or species, yet resolving this question is critical for accurate model predictions of carbon and water fluxes.

Stomata regulate CO_2_ and water exchange, coordinating photosynthetic carbon fixation with transpiration water loss (Cowan and Farquhar, 1977). Under non-stressed conditions, the close coupling between *g*_s_ and *A* underpins classic stomatal models like the Ball-Berry (BB) and unified stomatal optimization (USO) models (Ball et al., 1987; Medlyn et al., 2011). In the USO model, the slope parameter *g*_1_ reflects stomatal behavior: a higher *g*_1_ typically indicates “profligate” (lower water-use efficiency), lower *g*_1_ denotes more “conservative” behavior, i.e., stricter stomatal closure under high vapor pressure deficit (VPD) or progressive drought, leading to higher intrinsic water-use efficiency and lower hydraulic risk (Medlyn et al., 2011; Lin et al., 2015). Optimal stomatal theory suggests plants would downregulate *g*_1_ to maximize water-use efficiency under drought or high VPD (Manzoni et al., 2011; Zhou et al., 2013). During early drought, rapid stomatal closure limits CO_2_ entry and primarily limits *A* (Maroco et al., 2002; Grassi and Magnani, 2005; Guo et al., 2025). As drought intensifies, declining *A* becomes increasingly driven by non-stomatal limitations, including reduced mesophyll conductance (*g*_m_) and biochemical impairment (e.g., decreased Rubisco activity and photosystem II efficiency) (Flexas et al., 2004; Galmés et al., 2007; Salmon et al., 2020).

Warming alone has been extensively studied for its effects on leaf photosynthesis and chlorophyll fluorescence. Experimental warming can enhance, suppress, or negligibly influence net CO_2_ assimilation depending on species and the magnitude of warming (Way and Yamori, 2014; Dusenge et al., 2023; Hammer et al., 2025). In well−watered conditions, moderate warming typically increases both *g*_s_ and photosynthesis, but supra−optimal temperatures lead to declines due to stomatal and non−stomatal limitations (Scafaro et al., 2023). Under severe heat stress, warming often reduces Rubisco activity, damages photosystem II, and accelerates photorespiration (Crafts-Brandner and Salvucci, 2000; Dusenge et al., 2019). While some studies have examined warming-drought interactions, most have focused on short−term or severe drought rather than on progressive drought (Urban et al., 2017; Marchin et al., 2023), and fewer have quantified how warming alters the *g*_s_–*A* coupling parameters in stomatal models.

**Figure 1:**
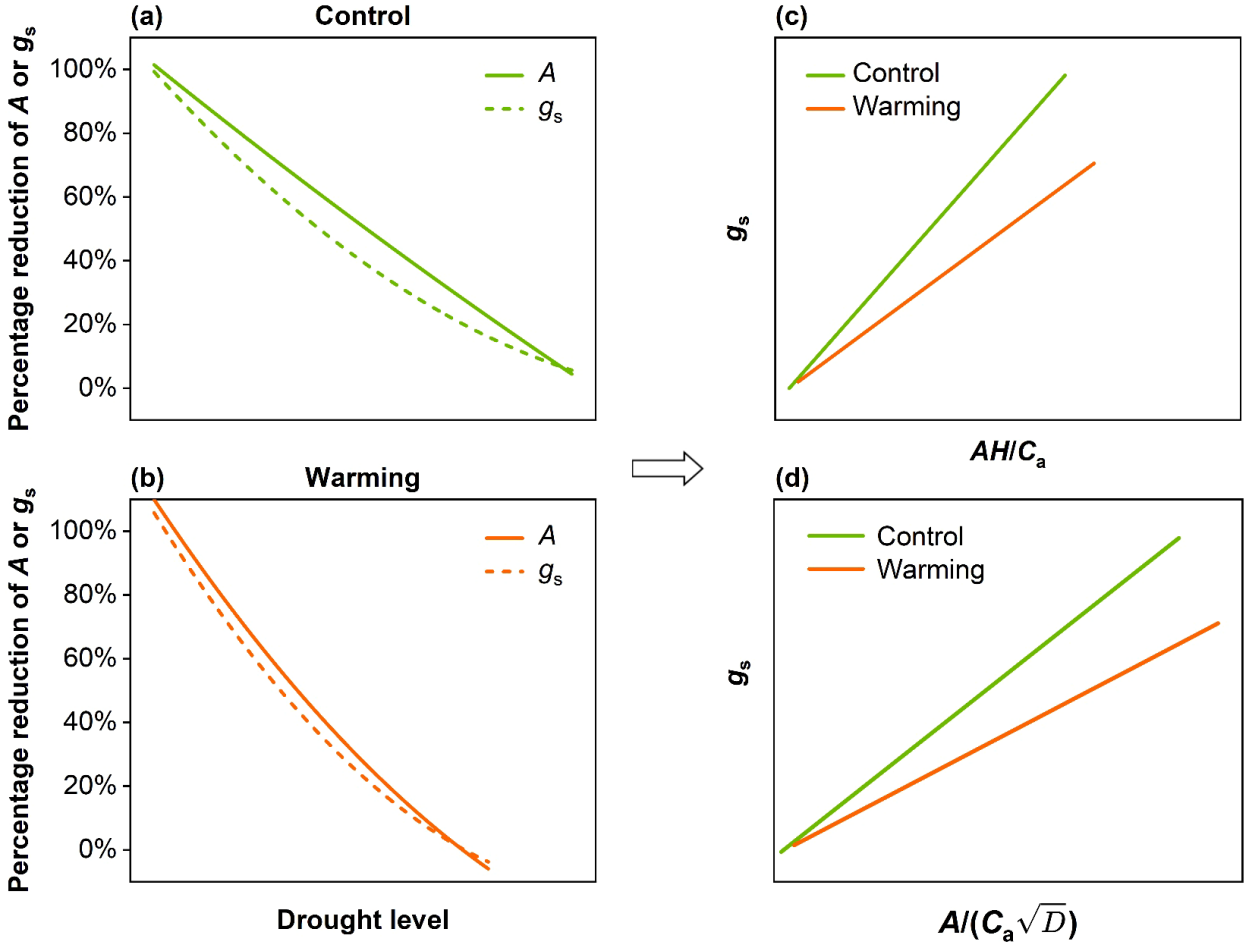
Conceptual diagram for variations in stomatal conductance (*g*_s_) and net assimilation rate (*A*) under control **(a)** and warming **(b)** conditions, and the relationship between *g*_s_ and *AH/C*_a_ **(c)**, the Ball-Berry model] and $A/\left(C_{\text{a}}\sqrt{D}\right)$ **(d)**, the unified stomatal optimization model] along progressive drought. We hypothesize that warming would cause a faster decline of *g*_s_ and *A* along progressive drought and decrease the slope parameter of the Ball-Berry and unified stomatal optimization models.

Growth temperature can modulate how *g*_s_ and *A* respond to decreasing soil moisture. Warming combined with drought typically increases stress by accelerating soil drying and raising VPD (Trenberth et al., 2014; Allen et al., 2015). To avert hydraulic failure, timely stomatal closure may push plants toward a more conservative stomatal behavior (Martin-StPaul et al., 2017; Choat et al., 2018) (**Figure 1**). Several studies have indeed found that warming increased stomatal sensitivity to drought and lowered *g*_1_ (Reich et al., 2018; Stefanski et al., 2023). Yet other studies reported that plants sustain relatively high *g*_s_ under combined warming and drought, presumably to cool leaves via transpiration (Drake et al., 2018; Marchin et al., 2022). These contrasting outcomes probably reflect differences in warming intensity, drought severity, and species-specific strategies. Indeed, direct species comparisons under combined warming and drought have revealed clear interspecific variation (e.g., Marchin et al., 2022), indicating different priorities among species regarding avoiding hydraulic failure and preventing overheating.

Interspecific variation in stomatal responses to environmental change is likely linked to leaf functional traits and underlying carbon-water tradeoffs (Medlyn et al., 2011; Lin et al., 2015; Miner et al., 2017). Under non-stressed conditions, species with lower leaf mass per area (LMA) and higher leaf nitrogen content generally achieve higher photosynthetic potential (Reich et al., 1997; Wright et al., 2004), as their nitrogen resources are prioritized for Rubisco and electron transport systems rather than protective mechanisms (Poorter et al., 2009; Onoda et al., 2017). This high metabolic demand is usually associated with higher *g*_s_ (Wong et al., 1979; Reich et al., 1999). However, thinner leaves render *g*_m_ vulnerable to dehydration, because dehydration-induced leaf shrinkage and changes in airspace structure can increase resistance to CO_2_ diffusion (Flexas et al., 2012; Scoffoni et al., 2014). In contrast, species with thicker leaves typically have greater structural stability and photoprotection capacity (Brodribb et al., 2014; Veromann-Jürgenson et al., 2017). While their potential photosynthetic capacity is lower, they maintain more stable *g*_m_ and use water conservatively, leading to higher intrinsic water-use efficiency (iWUE) (Brodribb et al., 2014; Wu et al., 2020). Still, leaf structural and nutrient traits do not determine stomatal behavior in isolation; the slope parameters (*m*, *g*_1_) in BB and USO models likely integrate the effects of gas exchange, biochemical capacity, and water status (Lin et al., 2015; Miner et al., 2017; Henry et al., 2019; Gardner et al., 2023). Exactly how these traits together shape stomatal behavior under the combined influence of warming and progressive drought remains an open question.

This study aimed to address two key questions regarding subtropical evergreen tree species: (i) How does warming alter stomatal behavior (quantified by the empirical slope parameter *m*_1_, see detailed in section 2.5.2) during progressive drought? (ii) Do different species exhibit divergent stomatal behavior under these combined stressors? To address these questions, we selected three dominant subtropical evergreen tree species native to southern China that occupy contrasting hydraulic strategies: *Castanopsis carlesii* is relatively vulnerable to hydraulic dysfunction under drought, *Schima superba*, possesses high xylem embolism resistance (Duan et al., 2019), and *Cunninghamia lanceolata* relies on high hydraulic safety margins and efficient leaf water storage (Ren et al., 2024; Xiang et al., 2026). Despite these reported differences in hydraulic traits, no study has compared their *g*_1_ dynamics under the joint pressure of warming and progressive drought. We conducted a controlled experiment to simulate warming combined with progressive drought. Dynamic measurements of leaf gas exchange and chlorophyll fluorescence were performed for all three species. Using both the BB and USO models, we quantified stomatal behavior in response to the warming−drought interaction. We hypothesize that: (H1) warming will cause a decline in *m*_1_ (i.e., a shift toward more conservative behavior) across all three species; and (H2) the species with stronger drought resistance (*S. superba*) will display stricter baseline stomatal regulation (lower *m*_1_) and a more rapid downward adjustment of *m*_1_ during progressive drought.

## 2 Materials and methods

### 2.1 Plant materials and growth conditions

In April 2024, seedlings of *C. carlesii*, *C. lanceolata*, and *S. superba* were transplanted into 19 L pots (top diameter: 28 cm, height: 31 cm) filled with a 1:1 mixture of light substrate soil (primarily composed of crushed bark, supplemented with approximately 20% peat moss and 10% perlite) and local sandy soil, with one seedling per pot. Considering the specific growth rates of each species, we used two-year-old *C. carlesii* and one-year-old *S. superba* and *C. lanceolata* seedlings. One-year-old seedlings of *S. superba* and *C. lanceolata* in this region are already of comparable size and physiological competence to two-year-old *C. carlesii*. Each pot received 15 grams of slow-release compound fertilizer (Dewoduo Controlled-Release Compound Fertilizer, Dewoduo, Hebei, China). Pots were then randomly arranged on a movable seedbed (15 m × 1.7 m) within a rainout shelter (detailed in **Supplementary Note 1**) at Yongtai Nursery, Fujian, China. The rainout shelter avoided the influence of precipitation on our experiment, allowing for precise control of soil moisture dynamics. Pest and weed management followed local standard procedures.

Warming treatments were applied from July 2024 to January 2025 with two temperature regimes: control (ambient) and warming (ambient +1°C). This warming magnitude aligns with the near-term (2021–2040) climate projections (IPCC, 2021). Passive warming was achieved using an open-top chamber (OTC) constructed of highly transparent acrylic resin. The OTC measured 70 cm in height and 50 cm in diameter at the base, tapering to 30 cm in height and 30 cm in diameter at the top. Each OTC contained one seedling, with four replicates per species for each temperature treatment (**Supplementary Figure 1**). The plants of control treatments were grown within the same rainout shelter without using OTCs. All plants were well-watered before the drought started, and the soil water content in control and warming treatments was not significantly different (**Supplementary Figure 2**). Progressive drought treatments began in December 2024, when irrigation was completely withheld until photosynthesis approached zero, marking the end of the drought experiment.

Air temperature and humidity were recorded using a temperature/humidity recorder (GSP-8, Jingchuang, Nanjing, China). Photosynthetically active radiation (PAR) was measured using a quantum sensor (SQ-202X-SS, Apogee Inc., Logan, USA) connected to a data collector (DJ-6095, Dianjiangtech, Shanghai, China). Soil water content (SWC) was measured using a portable soil moisture tester (ML3-KIT, Delta-T, Cambridge, UK). Temperature, humidity, and PAR data were recorded every five minutes. Environmental conditions during the experiment are summarized in **Supplementary Table 1** and **Supplementary Figure 1**. Daytime air temperature (mean daily value from 6 a.m. to 6 p.m.) averaged 25°C in the control treatment and 26°C in the warming treatment. Mean daytime VPD was 0.98 kPa in the control and 0.92 kPa under warming. Mean daytime light intensity was 363 µmol m^-2^ s^-1^, and the CO_2_ molar fraction was 420 µmol mol^-1^. Relative humidity was 74% (daytime) and 89% (nighttime) in the control treatment, and 77% (daytime) and 96% (nighttime) under warming. Overall, mean daytime VPD did not differ significantly between the control and warming treatments. Thus, the warming effect of our experiment is unlikely to be associated with elevated atmospheric drought.

### 2.2 *A-C_i_* curve and chlorophyll fluorescence measurements

Gas exchange measurements were performed using an open gas exchange system (Li-Cor 6800, LI-COR Inc., Lincoln, USA), with a fluorometer chamber with an adjustable aperture based on tree type: 6 cm^2^ for broadleaf trees or 2 cm^2^ for conifers. Measurements were conducted on the youngest fully expanded leaves. An initial baseline measurement was conducted on all plants while they were well-watered, before the onset of the drought treatment. Subsequent measurements were taken at seven-day intervals. Before *A*-*C*_i_ curves measurement, plants were dark-adapted for at least 30 min, after which leaf dark respiration (*R*_Dk_) was measured. *A*‐*C*_i_ response curves were measured under controlled conditions: a photosynthetic photon flux density (PPFD) of 480 μmol m^-2^ s^-1^ (provided by a 94% red/6% blue light source), a chamber temperature matching the respective treatment (25°C for control, 26°C for warming), and a relative humidity of 70% ± 10%. This PPFD of 480 μmol m^-2^ s^-1^ was selected because 1) it slightly exceeded the average growth PPFD, and 2) preliminary light-response curve measurements on *C. lanceolata* showed that the net assimilation rate at this PPFD reached 85 ± 4% of the light-saturated rate, confirming its suitability without causing photoinhibition. A uniform PPFD of 480 μmol m^-2^ s^-1^ was used for all species to ensure comparability. Under such operating conditions, *A*‐*C*_i_ curves enabled the calculation of the maximum rates of Rubisco carboxylation (*V*_cmax_) and the potential electron transport rate at a PPFD of 480 μmol m^-2^ s^-1^ (*J*_480_), which can act as a proxy for *J*_max_ (maximum electron transport rate) (Buckley and Diaz‐Espejo, 2015). After stabilization at the ambient CO_2_ mole fraction (*C*_a_, 420 μmol mol^-1^), gas exchange and fluorescence parameters were recorded at a sequence of *C*_a_ levels: 50, 100, 200, 420, 600, 800, 1000, and 1800 μmol mol^-1^. At each CO_2_ level, measurements were manually logged once photosynthetic rate and stomatal conductance had stabilized. *V*_cmax_ and *J*_480_ were estimated by using the fitting tool developed by Sharkey et al. (2007). Immediately after each gas exchange measurement session, the measured leaves were excised, and the fresh weight was measured using an electronic balance (ME204TE/02, Mettler-Toledo Instruments (Shanghai), Shanghai, China). The leaf area was scanned using a leaf area meter (YMJ-CHA3, Hangzhou, China), followed by freezing at -20°C. After freeze-drying the frozen leaf samples (FD-1A-50, Biocool, Beijing, China), the dry weight was measured, and the nitrogen mass fraction (N%) was determined using a CN elemental analyzer (Vario EL III, Elementar Analysensysteme GmbH, Hanau, Germany).

### 2.3 Estimating *g*_m_ using variable *J* method

The functional relationship (Equation 1) between electron transport rate of the carboxylation reaction (*J*), the net photosynthetic rate (*A*), and CO_2_ concentration in the carboxylation site of Rubisco (*C*_c_) in the Farquhar model (Farquhar et al., 1980) is combined with Fick’s law of diffusion (*C*_c_ = *C*_i_ – *A*/*g*_m_) to obtain *g*_m_ estimation equation:

(1) $$g_{\text{m}}=\frac{A}{C_{i}-\frac{\Gamma^{*}\left(J+8\left(A+R_{L}\right)\right)}{J-4\left(A+R_{L}\right)}}$$

*J* can be obtained based on the quantum yield of PSII measured by chlorophyll fluorescence (Equation 2), denoted as the electron transfer rate (Genty et al., 1989), with the following equation:

(2) $$J=\alpha \beta PPFD\Phi_{\text{PSII}}$$

where *α* is the leaf-activated light absorption coefficient (0.84), *β* is the proportion of activated light allocated to PSII (0.5), and *Φ*_PSII_ is the PSII photochemical efficiency, calculated from chlorophyll fluorescence parameters (Equation 3) (Genty et al., 1989):

(3) $$\Phi_{\text{PSII}}=\frac{F_{\text{m}}^{\prime }-F_{\text{s}}}{F_{\text{m}}^{\prime }}$$

where *F*_s_ is the steady state fluorescence in the light conditions, and $F_{\text{m}}^{\prime }$ is the maximal fluorescence during a short saturating pulse of light under actinic light, which was measured using the multiphase flash method.

Day respiration rate (*R*_L_) was estimated as 0.9 *R*_Dk_ in this study, based on the average *R*_L_*/R*_Dk_ value of 0.9 obtained using an advanced method for different species (Sun et al., 2023; Zheng et al., 2024). We acknowledge that the *R*_L_/*R*_Dk_ ratio can vary between species and environmental conditions (Crous et al., 2017; Heskel and Tang, 2018). Nonetheless, this assumption has little influence on the *g*_m_ estimates; sensitivity analysis shows that ±33% change of *R*_L_ results in an averaged ±8% change of *g*_m_ (**Supplementary Figure 3**). Г^*^ is the photorespiratory compensation point based on *C*_c_, estimated from leaf temperature (Equation 4) (Brooks and Farquhar, 1985):

(4) $$\Gamma^{*}=42.7+1.68\left(T-25\right)+0.012{\left(T-25\right)}^{2}$$

### 2.4 Quantifying the drought sensitivity of photosynthetic parameters

To quantify drought sensitivity, we introduced a slope-based metric termed the drought sensitivity index (DSI), defined as the slope of the linear regression between the relative decline ratio in photosynthetic parameters. This index captures the average daily rate of physiological decline and is calculated as (Equation 5):

(5) $$DSI=\frac{d\left(\frac{{\text{Y}}_{0}-{\text{Y}}_{\text{t}}}{{\text{Y}}_{0}}\right)}{dt}$$

where Y_0_ and Y_t_ represent the values of a photosynthetic parameter measured before and during progressive drought, respectively, and *t* represents the number of days of drought. When physiological responses did not exhibit a consistent linear trend over the progressive drought period, the fitted slopes were not statistically significant, indicating that the corresponding DSI was not significantly different from zero.

### 2.5 Parameterization of stomatal conductance models

#### 2.5.1 Ball-Berry model

The Ball‐Berry model (Equation 6) (Ball et al., 1987) describes the linear relationship between *g*_s_ and *AH*/*C*_a_:

(6) $$g_{\text{s}}=mA\frac{H}{C_{\text{a}}}+g_{0}$$

where *H* is the air relative humidity, and *m* and *g*_0_ are the slope and intercept, respectively. *m* represents the ‘compromise between costs and benefits of stomatal conductance relative to leaf photosynthetic activity’ (Collatz et al., 1991). *g*_0_ represents the minimum *g*_s_ (Ball et al., 1987; Collatz et al., 1991), or residual conductance at *A* ≤ 0 (Leuning, 1995). Our fitted results showed that values of *g*_0_ were close to zero in all species and thus were ignored.

#### 2.5.2 Unified stomatal optimization model

Medlyn et al. (2011) derived a model that approximates the estimation of optimal stomatal conductance:

(7) $$g_{\text{s}}=1.6\left(1+\frac{g_{1}}{\sqrt{D}}\right)\frac{A}{C_{\text{a}}}+g_{0}$$

Equation 7 predicts a close linear relationship between *g*_s_ and the combination of terms $A/\left(C_{\text{a}}\sqrt{D}\right)$ (Medlyn et al., 2011). And this linear relationship can be simplified by ignoring the intercept when its fitted values are close to zero (the case in our studied species):

(8) $$g_{\text{s}}=m_{1}\frac{A}{C_{\text{a}}\sqrt{D}}$$

Here, we use the simplified model (Equation 8) to simulate *g*_s_. The fitted slope *m*_1_ is empirically related to the theoretical optimization parameter *g*_1_. We therefore treat *m*_1_ as a proxy of stomatal behavior rather than a direct estimate of *g*_1_.

### 2.6 Limitation analysis

Relative quantitative limitations on assimilation were partitioned into stomatal conductance (*L*_s_), mesophyll conductance (*L*_m_), and biochemical limitations (*L*_b_), estimated using Equations 9–11 respectively, following the approach proposed by Grassi and Magnani (2005). The limitations of the different components were calculated as:

(9) $$L_{\text{s}}=\frac{\frac{g_{\text{tot}}}{g_{\text{sc}}}\times \frac{\partial A_{\text{n}}}{\partial C_{\text{c}}}}{g_{\text{tot}}+\frac{\partial A_{\text{n}}}{\partial C_{\text{c}}}}$$

(10) $$L_{\text{m}}=\frac{\frac{g_{\text{tot}}}{g_{\text{m}}}\times \frac{\partial A_{\text{n}}}{\partial C_{\text{c}}}}{g_{\text{tot}}+\frac{\partial A_{\text{n}}}{\partial C_{\text{c}}}}$$

(11) $$L_{\text{b}}=\frac{g_{\text{tot}}}{g_{\text{tot}}+\frac{\partial A_{\text{n}}}{\partial C_{\text{c}}}}$$

where *g*_sc_ is the stomatal conductance to CO_2_ (*g*_sc_=*g*_s_/1.6), *g*_tot_ is the total CO_2_ conductivity from ambient air to chloroplasts (1/*g*_tot_=1/*g*_sc_+1/*g*_m_). ∂*A*_n_/∂*C*_c_ was calculated as the slope of the *A*-*C*_c_ response curves, as follows (Tomás et al., 2013). But we differ by using a *C*_c_ range of 50-150 μmol mol^-1^ and *C*_i_ below 300 μmol mol^-1^ to ensure photosynthesis is limited by Rubisco activity, given the high CO_2_ saturation point in this study.

### 2.7 Statistical analysis

All data processing was conducted using Microsoft Excel (2016). Statistical analyses were performed with SPSS Statistics 19 (IBM Corp.). Specifically, we applied a two-way ANOVA to evaluate the main effects of warming, species, and their interactions on photosynthetic physiological parameters before the onset of drought. Where significant main effects or interactions were detected, *post-hoc* comparisons were performed using Tukey’s HSD test. Differences in the DSI of photosynthetic parameters across treatments and species were assessed using two-tailed *t*-tests. Linear regression analyses were conducted by pooling replicate data to derive overarching slopes (*m* and *m*_1_) across the drought period and were fitted with the LINEAR function in Origin 10.1 (Origin Pro 2024). Differences between fitted slopes were assessed using 95% confidence intervals based on the parameter covariance matrices of each linear model. Statistical significance was defined at *P* < 0.05 for all analyses. Since the measurements were taken repeatedly on the same individuals during the drought, we acknowledge that the data structure is hierarchical. While employing DSI and pooled regression to summarize individual trajectories, the potential non-independence of these observations is recognized as a limitation affecting the interpretation of statistical significance.

## 3 Results

### 3.1 Warming and progressive drought effects on gas exchange and leaf traits

**Figure 2:**
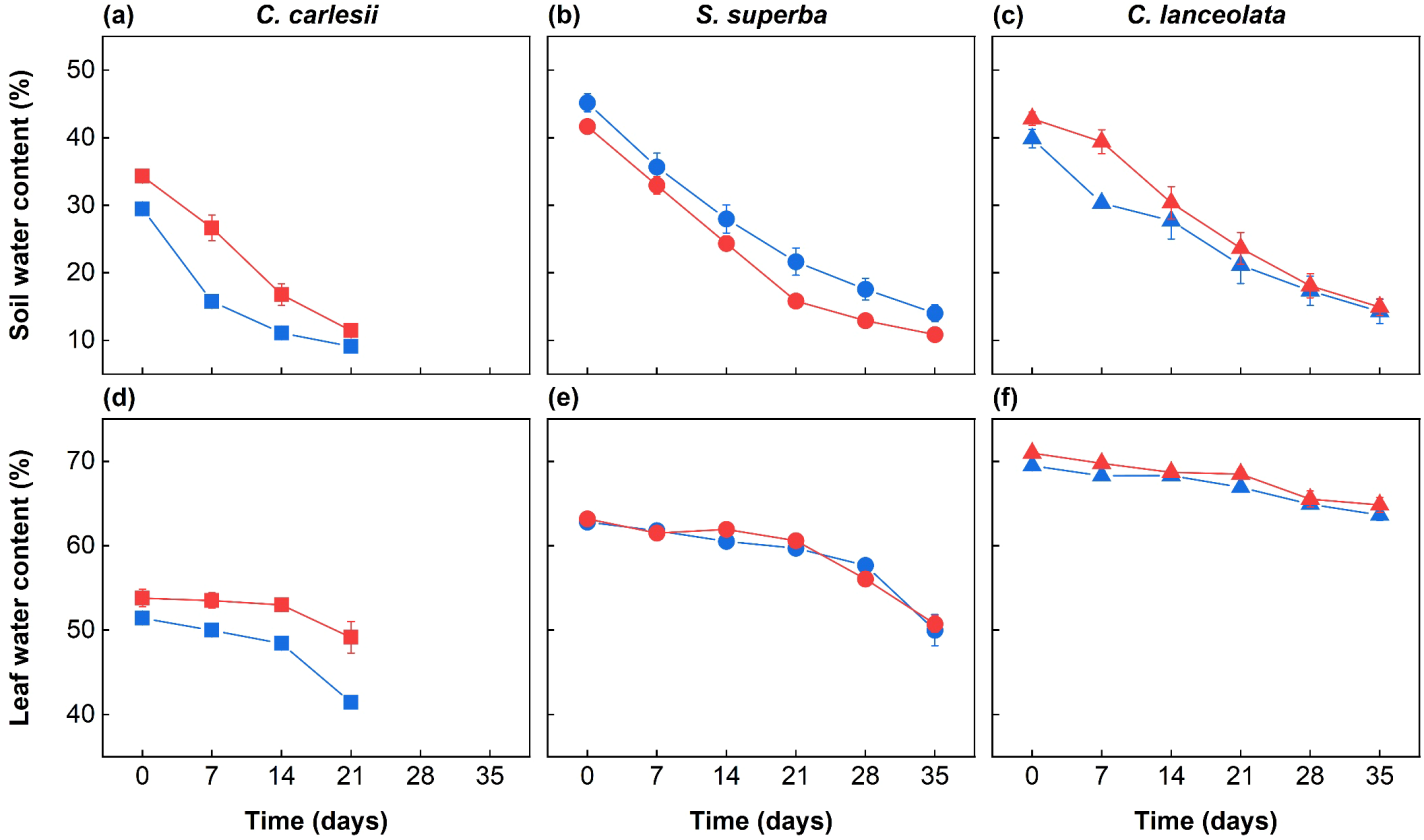
Soil water content (SWC) and leaf water content (LWC, (1-leaf dry mass/leaf fresh mass) × 100%) response to progressive drought of *C. carlesii* (panels **a, d**; square), *S. superba* (panels **b, e**; circle), and *C. lanceolata* (panels **c, f**; triangle) at control (blue) or warming (red). The first measurement (well-watered, day 0) was before progressive drought, after which watering was stopped to achieve progressive drought. Data are expressed as mean ± SE of each measurement (once in 7 days), n=3-4 (with the exceptions that n=1 for *C. carlesii* of control at day 21).

**Figure 3:**
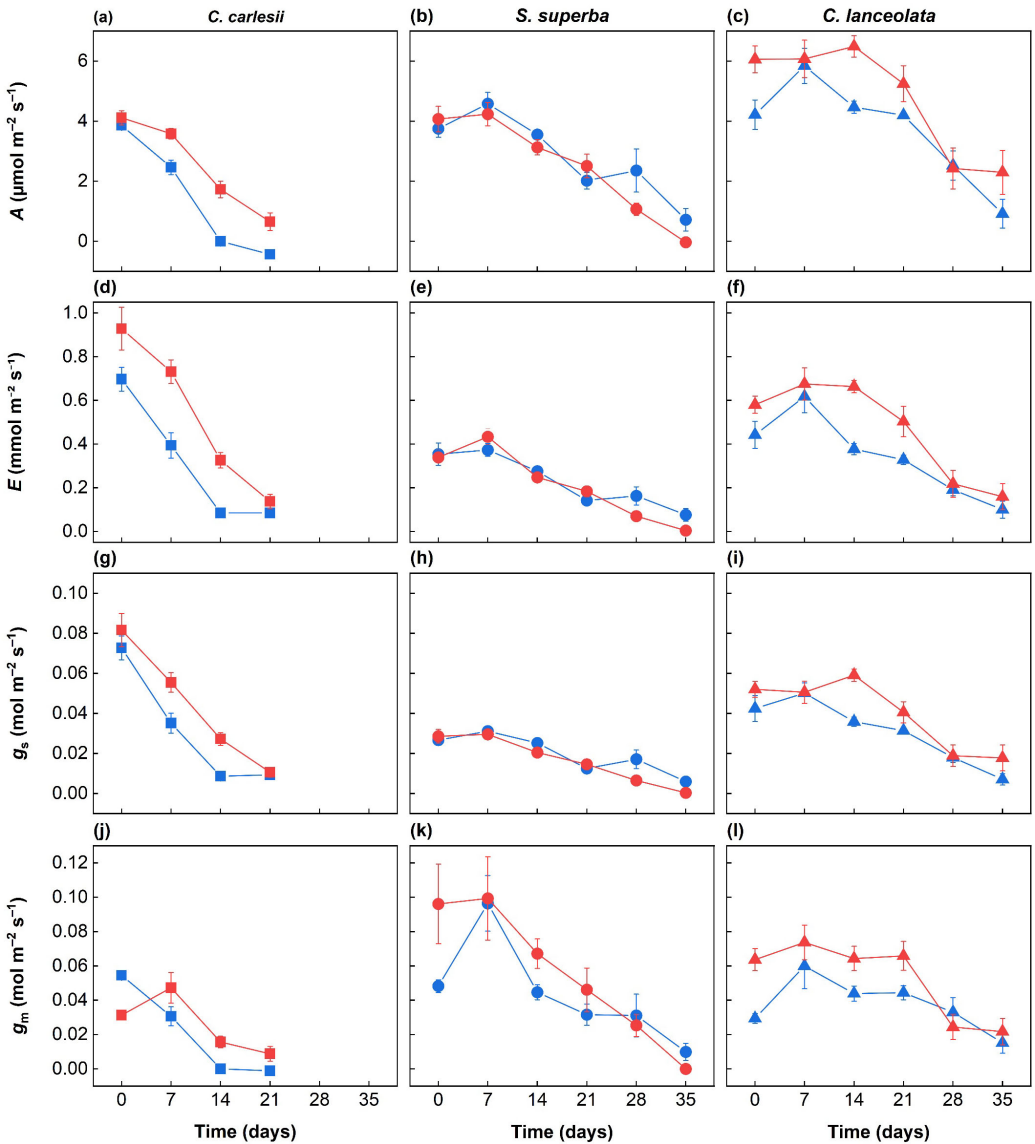
Net photosynthetic rate (*A*, μmol m^-2^ s^-1^), transpiration rate (*E*, mmol m^-2^ s^-1^), stomatal conductance to water vapor (*g*_s_, mol m^-2^ s^-1^), and mesophyll conductance (*g*_m_, mol m^-2^ s^-1^) at *C*_a_ of 420 μmol mol^-1^ response to progressive drought in *C. carlesii* (panels **a, d, g, j**; square), *S. superba* (panels **b, e, h, k**; circle), and *C. lanceolata* (panels **c, f, i, l**; triangle) at control (blue) or warming (red). The first measurement (well-watered, day 0) was before progressive drought, after which watering was stopped to achieve progressive drought. Data are expressed as mean ± SE of each measurement (once in 7 days), n=3-4 (with the exceptions that n=1 for *C. carlesii* of control at day 21).

**Figure 4:**
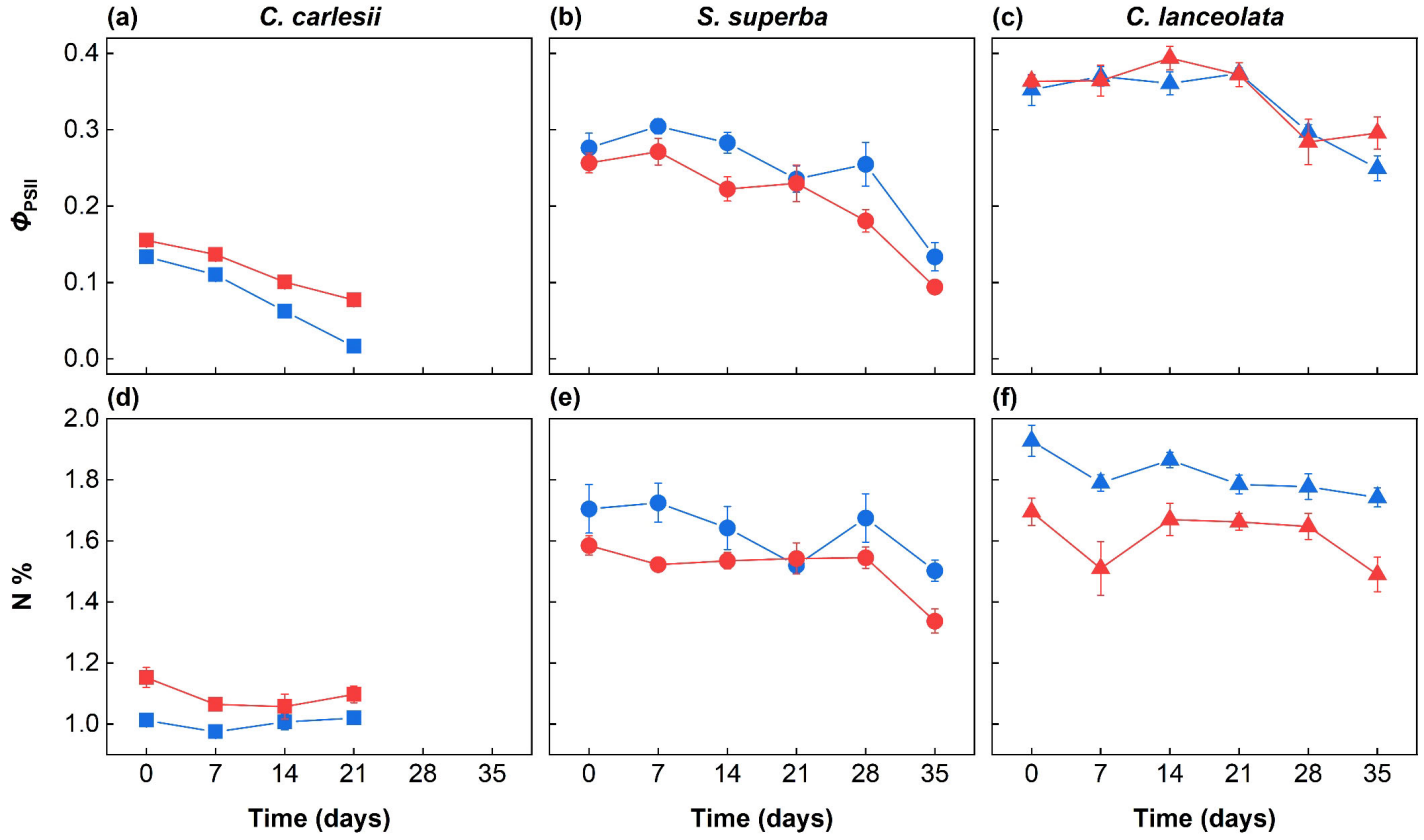
Photochemical efficiency of photosystem II (*Φ*_PSII_) at *C*_a_ of 420 μmol mol^-1^ and nitrogen mass fraction (N%) response to progressive drought of *C. carlesii* (panels **a, d**; square), *S. superba* (panels **b, e**; circle), and *C. lanceolata* (panels **c, f**; triangle) at control (blue) or warming (red). The first measurement (well-watered, day 0) was before progressive drought, after which watering was stopped to achieve progressive drought. Data are expressed as mean ± SE of each measurement (once in 7 days), n=3-4 (with the exceptions that n=1 for *C. carlesii* of control at day 21).

Under progressive drought conditions accompanied by warming, both SWC and leaf water content (LWC) progressively declined (**Figure 2**). Before drought imposition, plant height and ground diameter were not significantly influenced by warming (**Supplementary Figure 4**). *A*, transpiration rate (*E*), *g*_s_, *g*_m_, photochemical efficiency of photosystem II (*Φ*_PSII_), N%, LMA, iWUE, *V*_cmax_, and *J*_480_ were comparable between warming and control treatments for the three species (**Table 1**), except for *g*_m_ in *C. carlesii* and *C. lanceolata*. Notably, *g*_m_ under control conditions was +74% higher compared to warming for *C. carlesii*, whereas *C. lanceolata* exhibited +117% higher *g*_m_ under warming relative to control. When comparing tree species, *C. carlesii* had higher *E* and *g*_s_ compared to *S. superba* (+136% and +179%, respectively) and *C. lanceolata* (+59% and +64%, respectively) (**Table 1**; **Figure 3**), while its *Φ*_PSII_, N%, and iWUE were significantly lower than those of *S. superba* (-46%, -34%, and -61%) and *C. lanceolata* (-60%, -40%, and -51%) (**Table 1**; **Figure 4**; **Supplementary Figure 5**). No significant differences in *A*, *g*_m_, and *V*_cmax_ were observed across species (**Table 1**; **Figure 3**; **Supplementary Figure 6**).

**Table 1:** Comparison of warming effects and interspecific differences in photosynthetic parameters before of *C. carlesii*, *S. superba*, and *C. lanceolata* before progressive drought (well-watered, day 0).

	*C. carlesii*		*S. superba*		*C. lanceolata*		*P* values		
Parameters	Control	Warming	Control	Warming	Control	Warming	Treatments	Species	Tre×Spe
*A*	3.86 ± 0.1	4.11 ± 0.24	3.75 ± 0.29	4.07 ± 0.43	4.21 ± 0.49	6.06 ± 0.44	0.184	0.186	0.469
*E*	0.70 ± 0.05	0.93 ± 0.10	0.35 ± 0.05	0.34 ± 0.04	0.44 ± 0.06	0.58 ± 0.04	0.246	**0.004**	0.597
*g*_s_	0.073 ± 0.006	0.082 ± 0.008	0.027 ± 0.003	0.029 ± 0.003	0.042 ± 0.007	0.052 ± 0.004	0.458	**0.001**	0.928
*g*_m_	0.054 ± 0.001	0.031 ± 0.002	0.048 ± 0.004	0.096 ± 0.023	0.029 ± 0.003	0.064 ± 0.006	0.300	0.371	0.302
iWUE	56.24 ± 3.43	55.94 ± 5.40	144.92 ± 4.96	145.99 ± 5.12	110.15 ± 7.49	117.01 ± 0.95	0.758	**<0.001**	0.930
*Φ*_PSII_	0.13 ± 0.01	0.16 ± 0.01	0.28 ± 0.02	0.26 ± 0.01	0.35 ± 0.02	0.36 ± 0.01	0.846	**<0.001**	0.727
*V*_cmax_	29.18 ± 1.59	21.88 ± 0.88	44.51 ± 3.95	34.91 ± 4.22	43.15 ± 6.68	37.80 ± 3.84	0.271	0.139	0.965
*J*_480_	39.83 ± 2.13	31.66 ± 1.12	37.36 ± 1.99	46.65 ± 6.32	80.44 ± 6.66	78.73 ± 4.58	0.978	**<0.001**	0.610
N%	1.01 ± 0.01	1.15 ± 0.03	1.71 ± 0.08	1.59 ± 0.03	1.93 ± 0.05	1.70 ± 0.04	0.367	**<0.001**	0.155
LMA	84.05 ± 0.53	77.22 ± 1.86	69.41 ± 1.02	69.55 ± 2.12	70.43 ± 2.6	65.85 ± 2.27	0.237	**0.007**	0.646

Data are expressed as mean ± SE (n = 3-4). Significant differences are shown in bold (*P<* 0.05).

Net photosynthetic rate (*A*, μmol m^-2^ s^-1^), transpiration rate (*E*, mmol m^-2^ s^-1^), stomatal conductance to water vapor (*g*_s_, mol m^-2^ s^-1^), mesophyll conductance (*g*_m_, mol m^-2^ s^-1^), intrinsic water-use efficiency (iWUE, = *A*/*g*_s_, μmol mol^-1^), photochemical efficiency of photosystem II (*Φ*_PSII_), maximum carboxylation rate (*V*_cmax_, μmol m^-2^ s^-1^), potential electron transport rate at PPFD of 480 μmol m^-2^ s^-1^ (*J*_480_, μmol m^-2^ s^-1^), nitrogen mass fraction (N%), and leaf mass per area (LMA, g m^-2^).

During drought progression, *A*, *E*, and *g*_s_ significantly decreased across treatments, with the most pronounced reductions occurring on days 7 and 14 (**Figure 3**). The exception was *A* in control-treated *C. lanceolata*, which maintained stability before day 14. Significant *g*_m_ reductions occurred in control-treated *C. carlesii* and warming-treated *S. superba*. *Φ*_PSII_ declined significantly in both treatments of *C. carlesii* and *S. superba*, but remained stable in *C. lanceolata* before day 21 (**Figure 4**). N%, *V*_cmax_, and *J*_480_ showed no statistically significant responses to drought across species (**Figure 4**; **Supplementary Figure 6**).

### 3.2 Variations of photosynthetic drought sensitivity with warming and species

**Figure 5:**
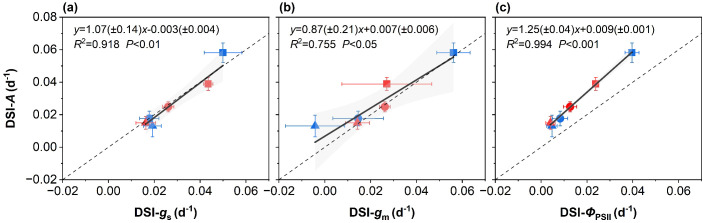
Relationships between drought sensitivity index (DSI)-*A* and DSI-*g*_s_ **(a)**, DSI-*g*_m_**(b)**, and DSI-*Φ*_PSII_ **(c)** of *C. carlesii* (square), *S. superba* (circle), and *C. lanceolata* (triangle) at control (blue) or warming (red). Each symbol represents the slope of drought response ratio ± SE (n=3-5).

During the progressive drought, *A* and *g*_s_ exhibited significant and robust linear declines across species and treatments (typically *R*^2^ > 0.7, *P* < 0.05; **Table 2**). However, the linear relationship was less consistent for *g*_m_ and *Φ*_PSII_. For instance, the linear fit for *g*_m_ in *C. carlesii* was relatively weak under warming (*R*^2^ = 0.232, *P* = 0.301), *g*_m_ and *Φ*_PSII_ showed non-significant linear responses in *C. lanceolata* (*P* > 0.05), and their DSI values (-0.004–0.014 and 0.004–0.005) were statistically nonsignificant. Warming did not significantly affect the DSI of *A*, *g*_s_, *g*_m_, and *Φ*_PSII_ in the studied species overall (**Table 2**; **Supplementary Figure 7**). When combining warming and control treatments, *g*_s_, *g*_m_, and *A* exhibited nearly identical DSI, whereas DSI-*Φ*_PSII_ decoupled from DSI-*A* (**Figure 5**). In terms of interspecific comparison, *C. carlesii* was more sensitive to drought, with higher DSI values for *A*, *g*_s_, and *Φ*_PSII_ exceeding those of *S. superba* (+143%, +124%, and +233%, respectively) and *C. lanceolata* (+253%, +164%, and +614%, respectively) (**Table 2**; **Supplementary Figure 7**). In contrast, DSI-*g*_m_ did not differ significantly among species, nor were there significant differences in any DSI parameters between *S. superba* and *C. lanceolata*.

**Table 2:** Drought sensitivity index (DSI) of photosynthetic parameters in two temperatures (Control and Warming) of *C. carlesii*, *S. superba*, and *C. lanceolata*.

Parameters	Control			Warming			Control×Warming		
	DSI (± CI)	*R* ^2^	*P*	DSI (± CI)	*R* ^2^	*P*	DSI (± CI)	*R* ^2^	*P*
*C. carlesii*									
*A*	0.058 ± 0.026	0.969	**0.01**	0.039 ± 0.017	0.969	**0.011**	0.049 ± 0.014	0.93	**<0.001**
*g*_s_	0.050 ± 0.035	0.923	**0.026**	0.044 ± 0.009	0.994	**0.002**	0.047 ± 0.01	0.957	**<0.001**
*g*_m_	0.056 ± 0.031	0.951	**0.016**	0.027 ± 0.084	0.232	0.301	0.042 ± 0.029	0.673	**0.015**
*Φ*_PSII_	0.04 ± 0.013	0.982	**0.006**	0.024 ± 0.006	0.99	**0.003**	0.032 ± 0.01	0.918	**<0.001**
*S. superba*									
*A*	0.018 ± 0.012	0.744	**0.017**	0.025 ± 0.008	0.93	**0.001**	0.021 ± 0.006	0.848	**<0.001**
*g*_s_	0.018 ± 0.012	0.772	**0.013**	0.026 ± 0.007	0.958	**<0.001**	0.022 ± 0.006	0.869	**<0.001**
*g*_m_	0.015 ± 0.031	0.128	0.258	0.026 ± 0.006	0.961	**<0.001**	0.02 ± 0.013	0.544	**0.006**
*Φ*_PSII_	0.008 ± 0.009	0.505	0.069	0.013 ± 0.008	0.786	**0.012**	0.01 ± 0.005	0.683	**0.001**
*C. lanceolata*									
*A*	0.013 ± 0.018	0.370	0.118	0.015 ± 0.011	0.732	**0.019**	0.014 ± 0.008	0.583	**0.004**
*g*_s_	0.019 ± 0.011	0.827	**0.008**	0.016 ± 0.012	0.731	**0.019**	0.018 ± 0.006	0.802	**<0.001**
*g*_m_	-0.004 ± 0.036	-0.217	0.759	0.014 ± 0.015	0.562	0.053	0.005 ± 0.016	-0.055	0.507
*Φ*_PSII_	0.005 ± 0.006	0.387	0.111	0.004 ± 0.006	0.368	0.119	0.004 ± 0.003	0.445	**0.015**

DSI (± CI) indicates drought sensitivity index ± confidence interval (Control, Warming, n=3-5; Control×Warming, n=6-10), *R*^2^ and *P* values are for linear fittings.

Net photosynthetic rate (*A*, μmol m^-2^ s^-1^), stomatal conductance to water vapor (*g*_s_, mol m^-2^ s^-1^), mesophyll conductance (*g*_m_, mol m^-2^ s^-1^), and photochemical efficiency of photosystem II (*Φ*_PSII_).

Significant differences are shown in bold (P < 0.05).

### 3.3 Species-specific coupling of stomatal conductance and photosynthesis

**Figure 6:**
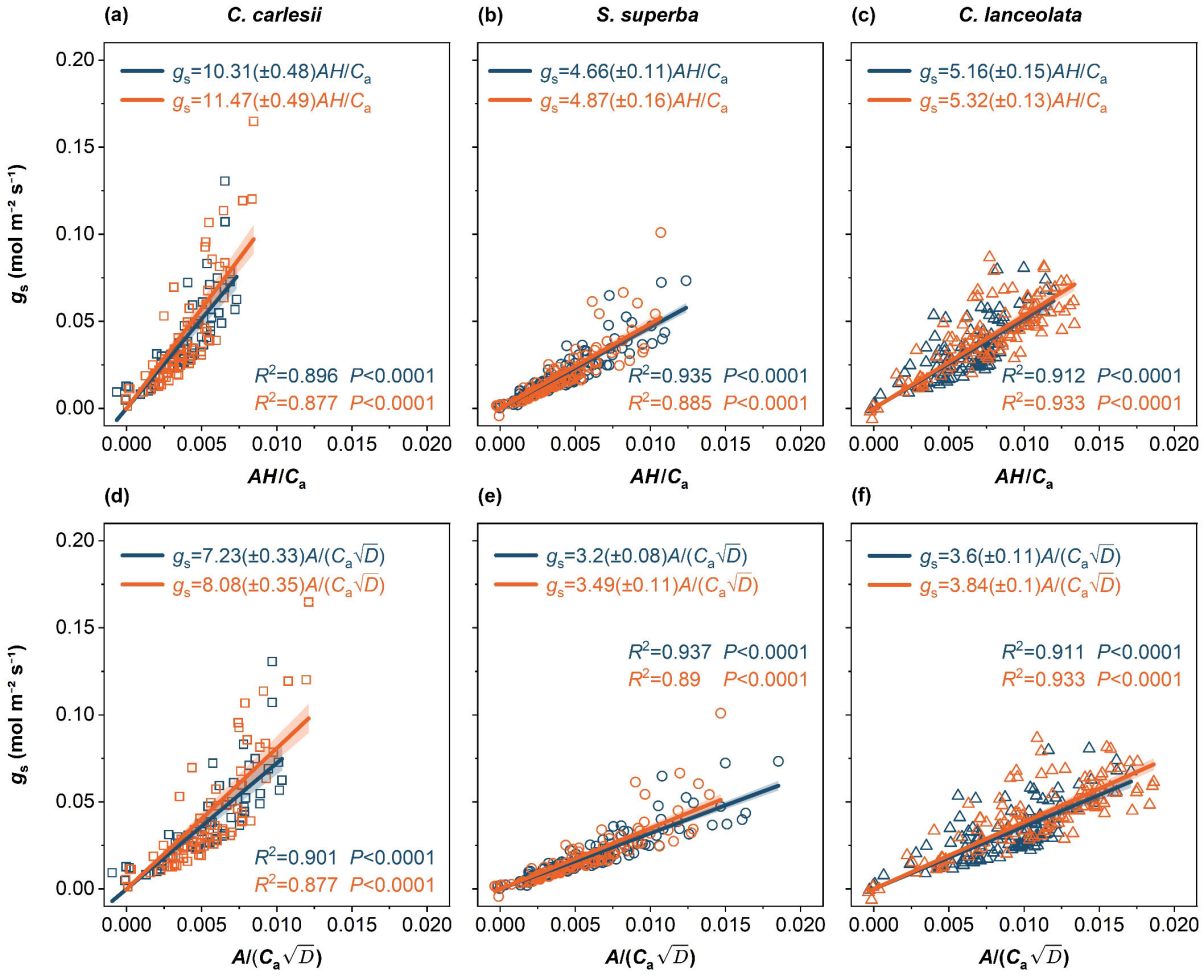
Relationships between *g*_s_ and *AH*/*C*_a_, $A/\left(C_{\text{a}}\sqrt{D}\right)$ of *C. carlesii* (panels **a, d**; number of measurements, n=129), *S. superba* (panels **b, e**; n=233), and *C. lanceolata* (panels **c, f**; n=227) at control (blue) or warming (orange) under progressive drought. Each symbol represents data points for individual plants at *C*_a_ concentrations of 200, 420, 600, 800, 1000, and 1800 μmol mol^–1^.

When fitting stomatal models, strong linear relationships were observed across all species and treatments (*R*^2^ > 0.87; **Figure 6**). While warming did not significantly influence the model parameters *m* (for the BB model) and *m*_1_ (for the simplified USO model), these values varied strongly across species. Both *m* and *m*_1_ were significantly higher in *C. carlesii* than in *S. superba* (+128% and +129%, respectively) and *C. lanceolata* (+108% and +106%, respectively) (**Figure 6**; **Supplementary Table 2**), and no significant interspecific differences were observed between *S. superba* and *C. lanceolata*. Additionally, intercept values (*g*_0_) approached zero in all three species for both stomatal models.

## 4 Discussion

### 4.1 Photosynthetic drought sensitivity is not affected by warming but tree species

Although photosynthesis significantly reduced with progressive drought, warming did not exacerbate photosynthetic drought sensitivity across the three studied species. This result is consistent with the findings of Aspinwall et al. (2022), who reported neutral effects of warming on gas exchange rates and unaltered drought response trajectories. Unlike the rapid photosynthetic collapse commonly observed in heat stress (Adams et al., 2015; Didion‐Gency et al., 2022), our study suggests a more homeostatic regulatory response. This context-dependent nature of warming effects has also been reported in subtropical conifers, where warming alone exerted limited influence on photosynthetic capacity, while drought strongly constrained temperature responses (Cui et al., 2026). The physiological mechanisms underlying photosynthetic reduction under drought include reduced *g*_s_ and/or *g*_m_ (Grassi and Magnani, 2005; Cano et al., 2014), inhibition of Rubisco activity (Galmés et al., 2011), and/or an impaired ribulose-1, 5-bisphosphate (RuBP) regeneration capacity (Thimmanaik et al., 2002). In this study, warming did not significantly affect the DSI of *g*_s_, *g*_m_, or *Φ*_PSII_, and consequently did not alter DSI-*A*. Collectively, these findings indicate that moderate warming does not necessarily amplify drought-induced photosynthetic inhibition, likely due to its minimal impact on plant water status and the functional integrity of the photosynthetic apparatus.

Pronounced interspecific variation in DSI reflected divergent hydraulic strategies and metabolic adjustments. *C. carlesii* exhibited the highest DSI, indicating a high vulnerability to hydraulic dysfunction under drought stress (Meinzer et al., 2016). Although *C. carlesii* possessed a higher LMA than the other species, its lower N% and iWUE suggest a reliance on higher *g*_s_ and lower *V*_cmax_ to maintain intercellular CO_2_ concentrations, thereby partially compensating for biochemical capacity limitations (Wright et al., 2004; Reich, 2014). This strategy may be advantageous under resource-rich conditions but likely increases vulnerability during prolonged drought (Reich, 2014). In contrast, *S. superba* demonstrated strict stomatal regulation and maintained higher leaf water content. Based on its previously documented robust xylem embolism resistance (Duan et al., 2019), *S. superba* may achieve a balance between carbon assimilation and hydraulic safety (Warren and Adams, 2006). Meanwhile, *C. lanceolata* maintained stable leaf water status and carbon assimilation even under severe drought, which, according to previous studies (Ren et al., 2024; Xiang et al., 2026), can be attributed to a high hydraulic safety margin, efficient leaf water storage capacity, and effective photoprotective mechanisms (Brodribb et al., 2014; Wu et al., 2020). These findings suggest that *S. superba* and *C. lanceolata* may possess greater physiological resilience than *C. carlesii* in subtropical China, a region projected to experience more frequent and severe droughts.

### 4.2 Stomatal conductance-photosynthesis coupling is maintained under warming and progressive drought

The stomatal-photosynthetic coupling remained robust across species under combined warming and progressive drought, indicating that reduced stomatal conductance largely regulates the decline in photosynthesis. Similar patterns have been documented in herbaceous species such as *Rhamnus alaternus* and *Rhamnus ludovici-salvatoris* (Bota et al., 2004) as well as in trees (Xu and Baldocchi, 2003; Héroult et al., 2013; Miner and Bauerle, 2017). A comparable pattern was also observed in the shrub *Artemisia ordosica* by Xu et al. (2025), where the decrease in carbon assimilation during early−summer drought was initially dominated by stomatal limitation, followed later by a reduction in leaf photosynthetic capacity. The synchronous decline in *E* and *g*_s_ (**Figure 3**) confirmed that the decrease in water flux was mainly limited by stomatal closure, with minimal contribution from cuticular conductance. However, the observed photosynthetic decline was not solely due to stomatal limitation. A close correlation between *g*_m_ and *A* was also evident (**Figure 5b**), highlighting the role of *g*_m_ in constraining CO_2_ diffusion to the chloroplasts (Flexas et al., 2013; Cano et al., 2014; Beauclaire et al., 2024). Furthermore, the slower decline in *Φ*_PSII_ relative to the reductions in *g*_s_ and *g*_m_ (**Figure 5**) aligns with findings from Wang et al. (2023). In contrast to previous studies reporting increased non-stomatal limitations to photosynthesis under drought (Grassi and Magnani, 2005; Galmés et al., 2007), our experiment did not detect significant shifts in the relative contributions of stomatal, mesophyll, or biochemical limitations (**Supplementary Figure 8**). The unchanged N% and *V*_cmax_ (**Figure 4**; **Supplementary Figure 6**) suggest that biochemical impairment was not a dominant factor under our experimental conditions. Nevertheless, we acknowledge that subtle biochemical adjustments, for instance in Rubisco activation state or RuBP regeneration capacity, could have occurred if a more severe or prolonged drought had been imposed. Despite the methodological challenges associated with measuring *g*_m_ (such as the indirectly determined *R*_L_), which resulted in greater data scatter *g*_m_ (Wang et al., 2024), stable relationships among *g*_s_, *g*_m_, and *A* were consistently observed across our experimental treatments. Our results revealed the coupling feature of *g*_s_ and *g*_m_ during progressive drought (**Supplementary Figure 5**), which is consistent with findings in other species (Ma et al., 2021; Márquez and Busch, 2024). Collectively, these results suggest that stomatal control played a dominant role in regulating photosynthesis during combined drought and warming.

Analysis of stomatal model slopes revealed distinct stomatal behavior among the studied species. The higher *m*_1_ in *C. carlesii* indicates a tendency to maintain higher *g*_s_ to ensure carbon uptake, albeit at the expense of reduced water use efficiency and increased drought risk (Medlyn et al., 2011; Manzoni et al., 2011). Correspondingly, this species experienced lower stomatal limitation on photosynthesis. In contrast, *S. superba* and *C. lanceolata* exhibited water-saving characteristics, reflected in their lower *m*_1_ values. This observation partly aligns with the global trend wherein gymnosperms generally possess a lower slope parameter than angiosperms (Lin et al., 2015; Miner et al., 2017). However, the low *m*_1_ in the angiosperm *S. superba* is likely more closely related to its specific hydraulic safety requirements than solely to phylogenetic constraints (Duan et al., 2019; Zhu et al., 2024).

### 4.3 Implication of the coupling between stomatal conductance and photosynthesis under warming and progressive drought

The tight coupling between *g*_s_ and *A* observed under warming and progressive drought in this study suggests that subtropical trees may exhibit substantial physiological resilience to near-term climate change. The warming magnitude in this experiment aligns with near-term climate projections and likely remains within the thermal optimum for photosynthesis of these subtropical species. This prevented the physiological decoupling or damage to the photosynthetic apparatus that is often induced by extreme heatwaves (Slot and Winter, 2017; Marchin et al., 2023). Such coupling enables plants to optimize carbon gain per unit of water loss, a key adaptive trait in dynamic, stress-prone environments. The stability of this relationship supports the use of optimality-based stomatal models for predicting GPP under near-term warming scenarios (Grossiord et al., 2017). However, pronounced interspecific variation in stomatal behavior and photosynthetic sensitivity highlights the necessity of incorporating species-specific parameters, particularly for projecting how species respond to warming and/or drought affect the land carbon sink, land–atmosphere feedbacks and shifts in species’ ranges (De Kauwe et al., 2015; De Kauwe et al., 2022). Accurately accounting for the differences in stomatal regulation among co-occurring species will substantially improve the predictive accuracy of models for forest carbon and water fluxes (Joshi et al., 2022; Mengoli et al., 2025).

It should be noted that these findings are derived from studies on seedlings under controlled conditions and may not be fully extrapolated to mature trees or more severe climate scenarios. Mature trees typically possess distinct hydraulic architectures, deeper root systems, and greater carbon reserves, all of which enhance their capacity to buffer against drought stress (Niinemets, 2010), traits that are not fully captured in seedling experiments. Specifically, the warming treatment applied here (approximately +1°C above ambient) represents a modest, near−term climate scenario rather than an extreme heatwave. Larger increases in temperature may trigger non−linear physiological responses, including irreversible damage to the photosynthetic apparatus and decoupling of the *g*_s_–*A* relationship. Therefore, our conclusion that warming did not exacerbate drought sensitivity should be interpreted within this moderate warming context and should not be directly extrapolated to more severe warming scenarios without further experimental validation. Furthermore, the absence of an interactive effect between warming and drought on physiological responses is likely related to the limited magnitude of warming in our experiment, which did not significantly alter soil moisture depletion patterns. Future studies should integrate whole-plant hydraulic traits, such as xylem vulnerability to embolism and root water uptake dynamics (Tng et al., 2018; Bhusal et al., 2025), along with higher warming gradients to evaluate the carbon-water coupling of trees.

We acknowledge a limitation in our experimental design: the warming treatment was applied using OTCs, while control plants were grown without OTCs. Although OTCs primarily elevated temperature and did not significantly alter daytime VPD (**Supplementary Table 1**), they may have introduced secondary microclimatic differences (e.g., reduced airflow, altered boundary layer conductance, and lower turbulence) compared to the open control conditions. Such factors can influence leaf transpiration and stomatal behavior independently of temperature. Therefore, because a chambered control treatment was not included, the observed treatment effects should be interpreted as the combined influence of moderate warming and OTC-associated microenvironmental changes rather than the effect of warming alone.

## 5 Conclusion

**Figure 7:**
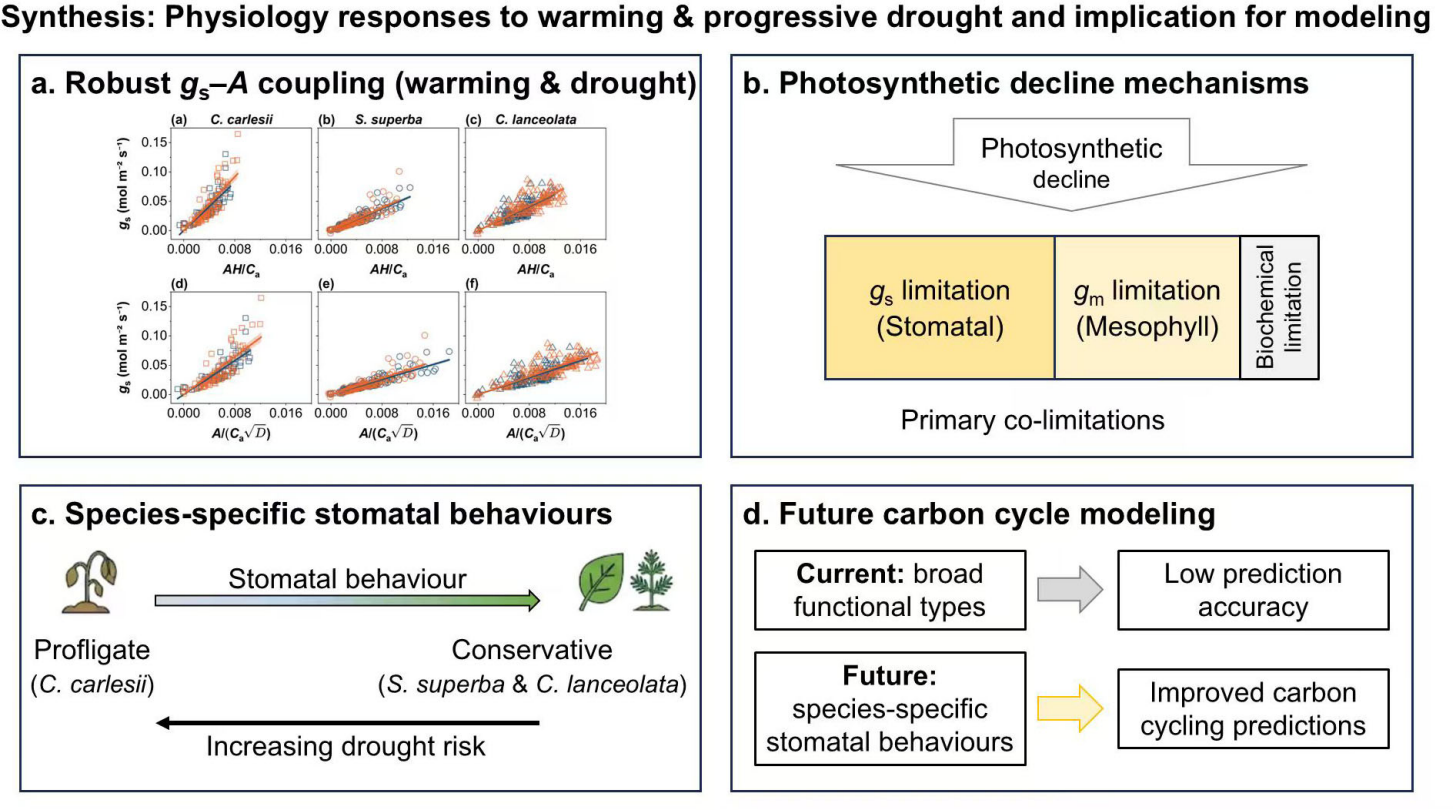
Summary of the effects of warming and progressive drought on the photosynthetic physiology of three subtropical tree seedlings and implications for modeling. **(a)** Robust *g_s_–A* coupling under warming and progressive drought, **(b)** underlying mechanisms for photosynthesis decline, **(c)** species-specific stomatal behaviours, **(d)** implications for future modeling.

Our study demonstrates that moderate warming (+1°C) did not alter photosynthetic drought sensitivity or disrupt *g*_s_–*A* coupling in subtropical seedlings during progressive drought (**Figure 7**), supporting the use of stomatal optimality models for the studied species under near-future climate scenarios. The observed photosynthetic decline was associated with concurrent reductions in *g*_s_ and *g*_m_. We identified distinct interspecific differences in stomatal behavior: the drought-sensitive *C. carlesii* exhibited a less conservative stomatal behavior, resulting in higher drought risk, whereas *S. superba* and *C. lanceolata* displayed more conservative stomatal behavior. Potentially, incorporating species-specific stomatal behavior may improve carbon−flux predictions by land surface models, particularly for plantations dominated by the species studied here.

## Data Availability

The raw data supporting the conclusions of this article will be made available by the authors, without undue reservation.
