# Supplementary material for: Coupling between stomatal conductance and photosynthesis of subtropical tree seedlings under warming and progressive drought
Source: Front Plant Sci. 2026 Jun 29;17:1846577. doi: 10.3389/fpls.2026.1846577 (PMC13357574; doi:10.3389/fpls.2026.1846577)
Supplement: Supplementary file 1 [file DataSheet1.pdf]

Note S1 Experimental facility and microclimate conditions.

The experiment was conducted within a rainout shelter at the Yongtai Nursery, Fujian, China. The shelter featured a semi-cylindrical galvanized steel frame covered with transparent polycarbonate sheets (light transmittance of approximately 34%). This structure effectively excluded natural precipitation, enabling precise control of soil moisture. To prevent heat accumulation in the rainout shelter, the sidewalls were kept open up to 2 m above the ground to facilitate ventilation. Microclimate variables in the shelter were continuously monitored from July 2024 to January 2025. The mean daytime (6 a.m.–6 p.m.) vapor pressure deficit (VPD) between the warming (1.30 kPa) and control (1.21 kPa) treatments was not significantly different (Table S1), indicating that the passive warming treatment did not impose atmospheric drought stress. The mean daily photosynthetically active radiation (PAR) inside the shelter was 363 μmol m^{-2} s^{-1}. While lower than natural outdoor conditions, this light environment broadly corresponds to forest understory or gap conditions and prevents potential photoinhibition or leaf overheating, which is suitable for the growth of seedlings.

Table S1 Air temperature, relative humidity and vapor pressure deficit (VPD) during daytime and nighttime inside (warming) and outside (control) the open-top chamber, and the averaged photosynthetic active radiation (PAR) from 6 a.m. to 6 p.m. each day before the start of drought. Significant differences between treatments are shown in different lowercase letters ($P < 0.05$).

	Warming (day/night)	Control (day/night)
Temperature (°C)	29^a / 23^a	28^a / 23^a
Relative humidity (%)	77^a / 96^a	74^b / 89^b
VPD (kPa)	0.92^a / 0.11^b	0.98^a / 0.31^a
PAR (μmol m^{-2} s^{-1})	363	363

Table S2 Comparison of warming effects and interspecific differences in slope parameters for Ball-Berry (*m*) and simplified USO models (m_1) of *C. carlesii*, *S. superba*, and *C. lanceolata* under progressive drought. Data are expressed as fitted slope ± CI (n=53-119). Significant differences between species are shown in different lowercase letters for each treatment ($P < 0.05$), and no significant differences between treatments for each species.

	C. carlesii		*S. superba*		*C. lanceolata*	
Parameters	Control	Warming	Control	Warming	Control	Warming
m	10.31±0.97[a]	11.47±0.98[a]	4.66±0.22[b]	4.87±0.33[b]	5.16±0.30[b]	5.32±0.26[b]
m_1	7.23±0.66[a]	8.08±0.69[a]	3.20±0.15[b]	3.49±0.23[b]	3.60±0.21[b]	3.84±0.19[b]

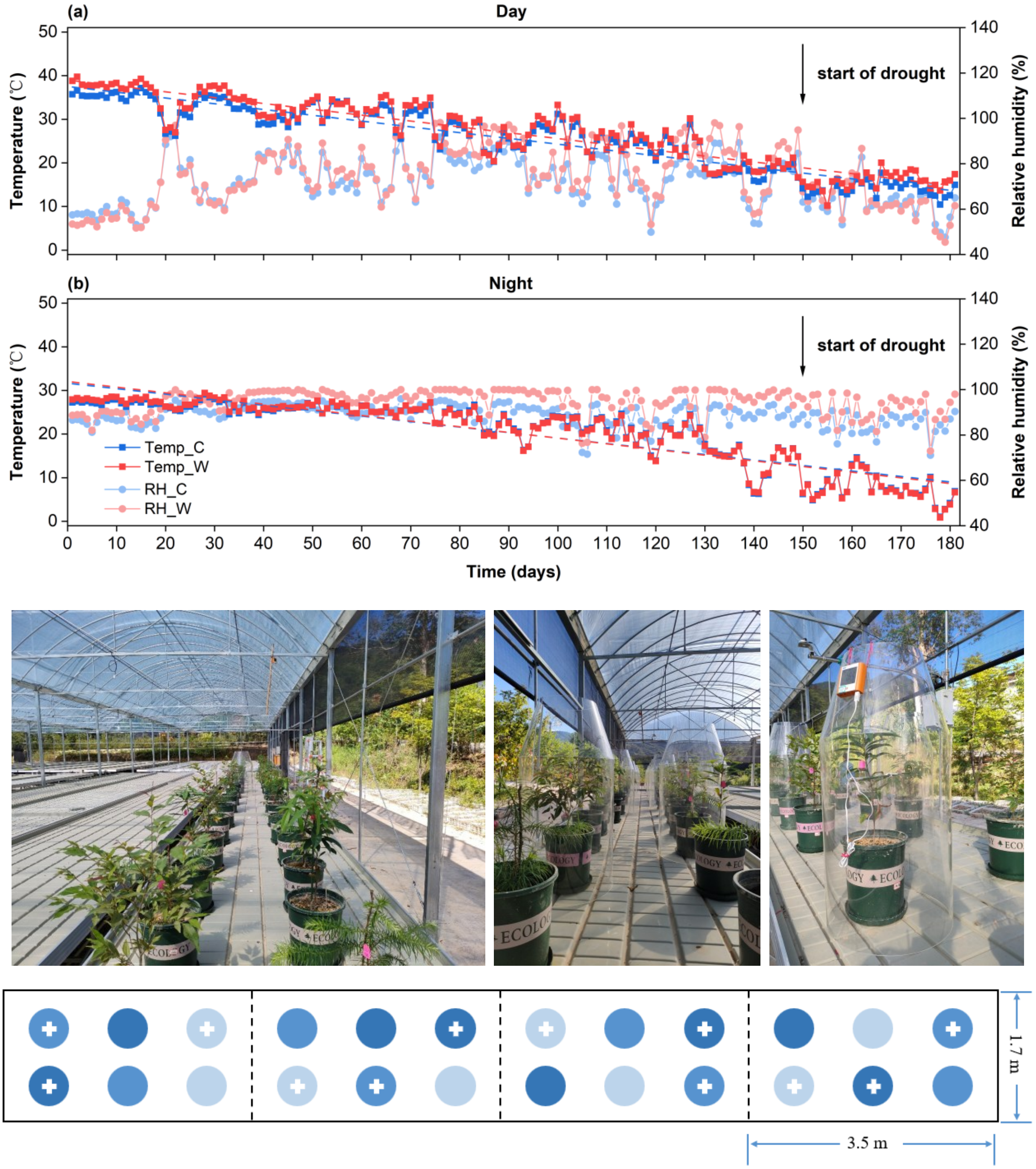

Fig. S1. Figure of air temperature and relative humidity variation, and schematic diagram of rainout shelter and open top chamber. Temperature and relative humidity variations for the control (blue) and warming (red), with (a) indicating daytime (from 6 a.m. to 6 p.m.) and (b) indicating nighttime from the onset of warming to the end of drought. The arrows indicate the start of drought imposition. Rainout shelter facilities, open-top chamber warming treatment, and potted plant placement. Colors from light to dark represent *Castanopsis carlesii*, *Schima superba*, and *Cunninghamia lanceolata*, respectively.

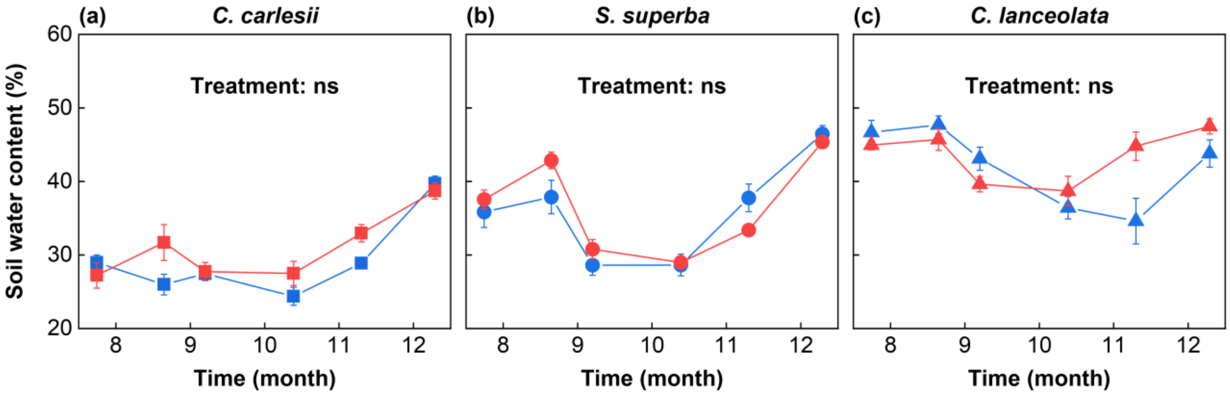

Fig. S2. Averaged soil water content (%) before the start of drought, which was measured on July 23, Aug 20, Sep 6, Oct 12, Nov 9, Dec 9 2025, in *C. carlesii* (square), *S. superba* (circle), and *C. lanceolata* (triangle) at control (blue) and warming (red). Data are mean ± SE (n = 3-4). There was no significant difference in soil water content between control and warming for all three species ($P > 0.05$).

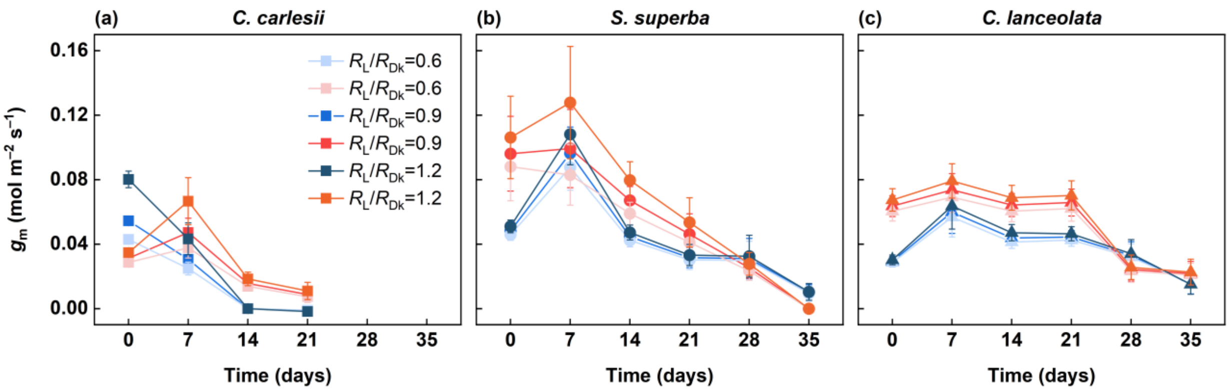

Fig. S3. Sensitivity analysis of g_m prediction by the variable-J method as affected by R_L/R_{Dk} ratios in *C. carlesii* (square), *S. superba* (circle), and *C. lanceolata* (triangle) at control (blue) and warming (red or orange). Data are mean ± SE (n = 3-4) (with the exceptions that n=1 for *C. carlesii* of control at day 21). Colours from light to dark represent R_L/R_{Dk} = 0.6, 0.9, and 1.2, respectively.

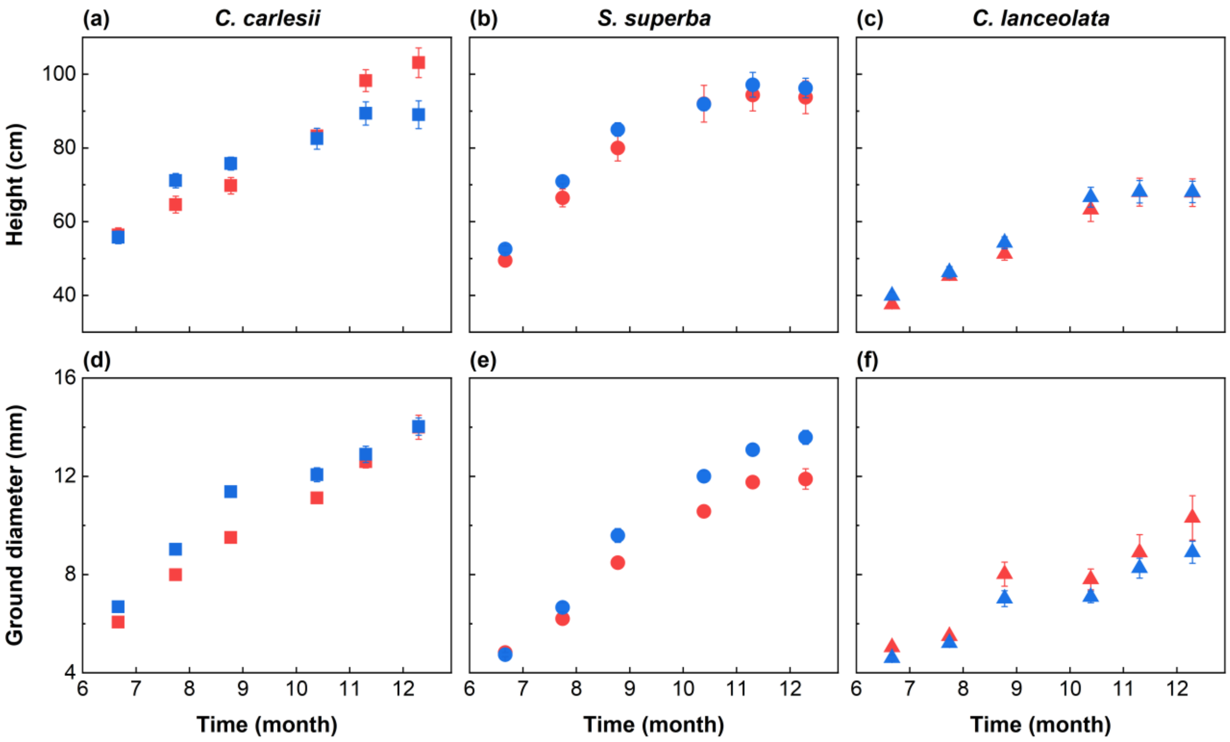

Fig. S4. Height (cm) and ground diameter (mm) in *C. carlesii* (square), *S. superba* (circle), and *C. lanceolata* (triangle) at control (blue) and warming (red). Data are mean ± SE (n = 4).

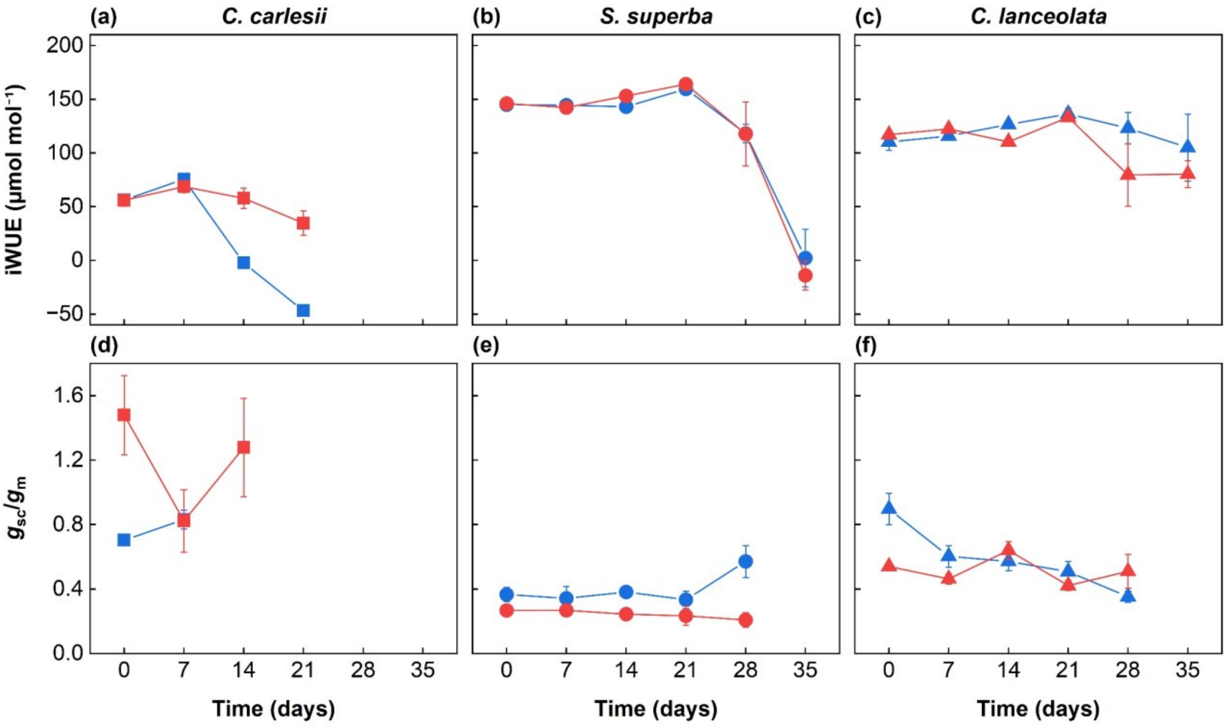

Fig. S5. Intrinsic water-use efficiency (iWUE = A/g_s) and stomatal conductance to CO_2 to mesophyll conductance ratio (g_{sc}/g_m) at C_a of 420 µmol mol^{-1} response to progressive drought in *C. carlesii* (square), *S. superba* (circle), and *C. lanceolata* (triangle) at control (blue) or warming (red). The first measurement (well-watered, day 0) was before progressive drought, after which watering was stopped to achieve progressive drought. Data are mean ± SE (n = 3-4) (with the exceptions that n=1 for *C. carlesii* of control at day 21), g_{sc}/g_m exclude negative g_{sc} or g_m.

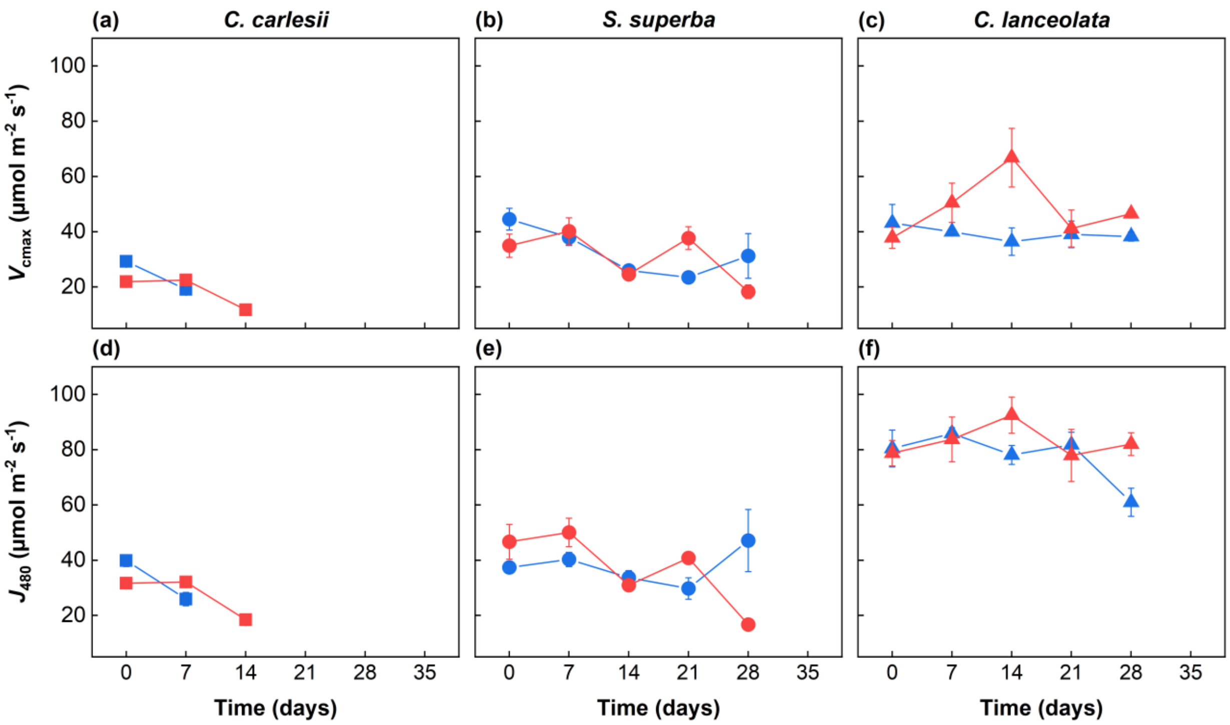

Fig. S6. Maximum carboxylation rate (V_{cmax}, μmol m^{-2} s^{-1}) and potential electron transport rate at PPFD of 480 μmol m^{-2} s^{-1} (J_{480}, μmol m^{-2} s^{-1}) response to progressive drought in *C. carlesii* (square), *S. superba* (circle), and *C. lanceolata* (triangle) at control (blue) or warming (red). Data are mean ± SE (n = 3-4). A-C_i curves of *C. carlesii* at the control at day 14 were not obtained.

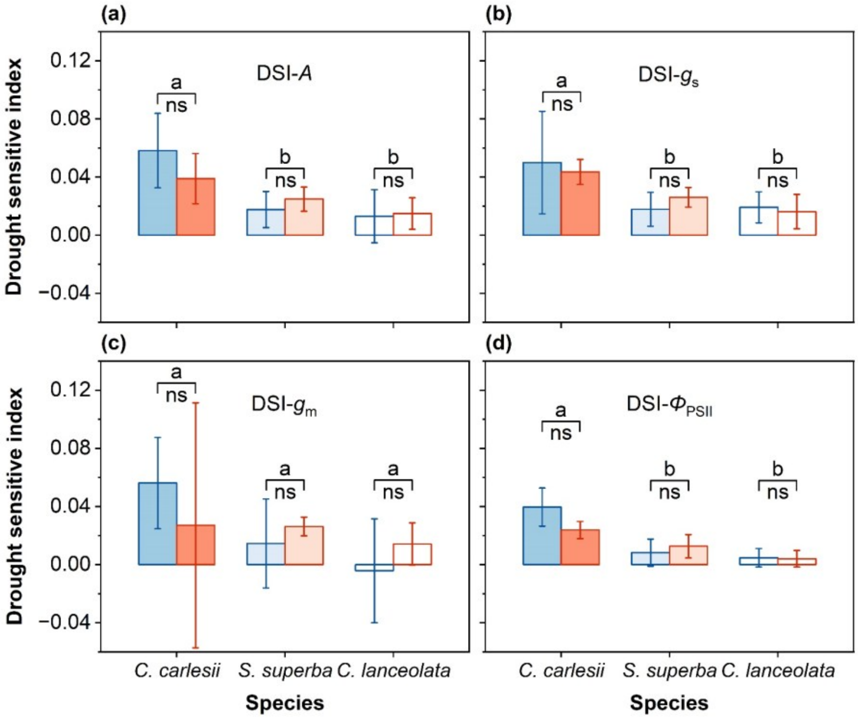

Fig. S7. Drought sensitivity index (DSI) of net photosynthetic rate (*A*), stomatal conductance to water vapor (g_s), mesophyll conductance (g_m), and photochemical efficiency of photosystem II (Φ_{PSII}). Blue and red represent control and warming, respectively, and from dark to light represent *C. carlesii*, *S. superba*, and *C. lanceolata*, respectively. Bars indicate slope of drought response rate ± CI (n=3-5), a, b indicate significant differences between species, and ns indicates insignificant.

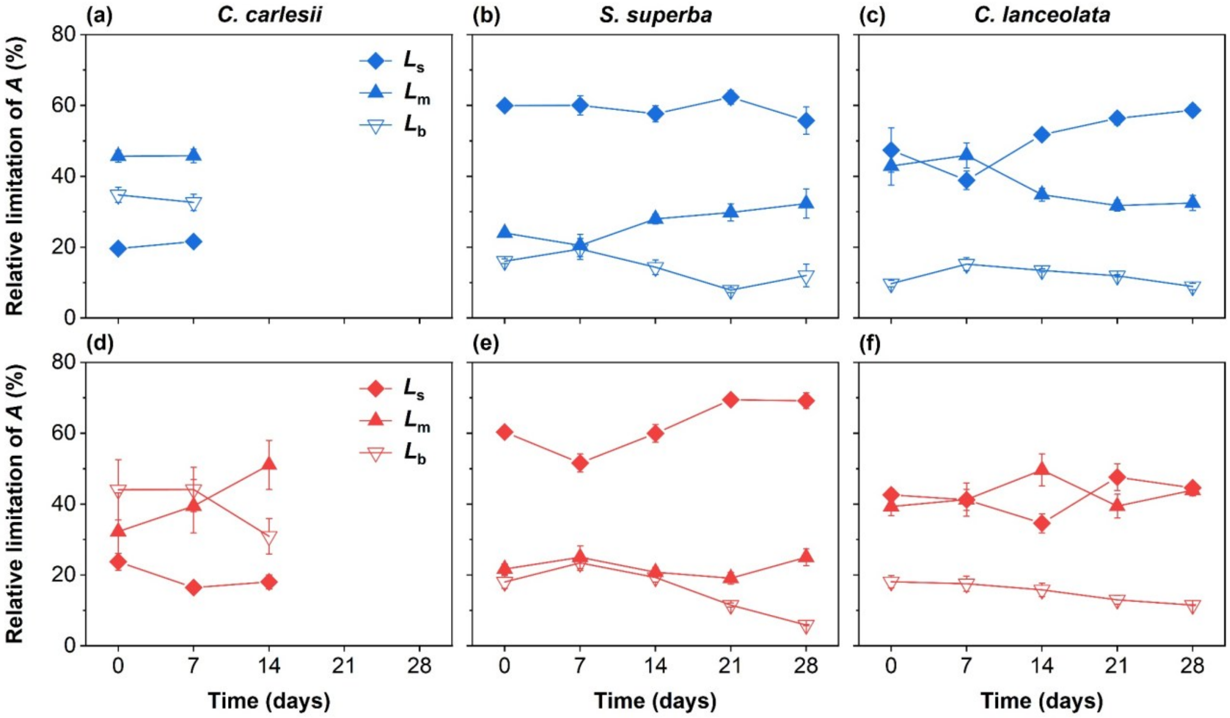

Fig. S8. Effect of progressive drought on relative limitation of *A* in *C. carlesii*, *S. superba* and *C. lanceolata* at control (blue) or warming (red). The first measurement (well-watered, day 0) was before progressive drought, after which watering was stopped to achieve progressive drought. Total photosynthetic limitation consists of stomatal (L_s), mesophyll conductance (L_m) and biochemical limitations (L_b). Data are mean ± SE (*C. carlesii*, n = 2-3, *S. superba* and *C. lanceolata*, n = 3-4).
